# CRISPR-based tools for plant genome engineering

**DOI:** 10.1042/ETLS20170011

**Published:** 2017-09-15

**Authors:** Nathalia Volpi e Silva, Nicola J. Patron

**Affiliations:** 1 Department of Plant Biology, Institute of Biology, University of Campinas (UNICAMP), Campinas, Brazil; 2 The Earlham Institute, Norwich Research Park, Norfolk NR4 7UZ, U.K.

**Keywords:** CRISPR, DNA assembly, genome editing, genome engineering

## Abstract

Molecular tools adapted from bacterial CRISPR (clustered regulatory interspaced short palindromic repeat) adaptive immune systems have been demonstrated in an increasingly wide range of plant species. They have been applied for the induction of targeted mutations in one or more genes as well as for directing the integration of new DNA to specific genomic loci. The construction of molecular tools for multiplexed CRISPR-mediated editing in plants has been facilitated by cloning techniques that allow multiple sequences to be assembled together in a single cloning reaction. Modifications of the canonical Cas9 protein from *Streptococcus pyogenes* and the use of nucleases from other bacteria have increased the diversity of genomic sequences that can be targeted and allow the delivery of protein cargos such as transcriptional activators and repressors. Furthermore, the direct delivery of protein–RNA complexes to plant cells and tissues has enabled the production of engineered plants without the delivery or genomic integration of foreign DNA. Here, we review toolkits derived from bacterial CRISPR systems for targeted mutagenesis, gene delivery and modulation of gene expression in plants, focusing on their composition and the strategies employed to reprogramme them for the recognition of specific genomic targets.

## Introduction

Bacterial CRISPR (clustered regulatory interspaced short palindromic repeat) regions consist of short-repeated sequences interspaced with non-homologous sequences, known as spacers, found to have been acquired from previously encountered pathogens [1]. When functioning in immunity, RNAs transcribed from the CRISPR locus (crRNAs), together with a *trans*-activating RNA (trRNA), are processed into guide RNAs (gRNAs), each with an individual spacer sequence. gRNAs form a complex with one or more CRISPR-associated (Cas) proteins to scan and cleave invading DNA at regions with similarity to previously acquired spacers [2]. The majority of genome engineering in eukaryotes to date has used tools adapted from the *Streptococcus pyogenes* CRISPR system in which the monomeric nuclease, Cas9, in complex with a gRNA, scans double-stranded DNA pausing at protospacer adjacent motifs (PAMs) with the canonical sequence NGG [3]. On recognition of a PAM, the spacer region of the gRNA is brought into proximity with the genomic DNA adjacent to the PAM and, if complementary, the nuclease domains of the Cas9 protein cleave both DNA strands. The induced break is repaired by the cell's endogenous repair mechanisms. When used for genome engineering, these breaks can be leveraged for targeted mutagenesis, where the exact mutation is not controlled but is the result of imperfect repair; for targeted delivery of new DNA sequences, or to recode the endogenous sequence to a desired sequence (commonly known as genome editing).

To direct Cas9 to a desired genetic locus, it is only necessary to recode the 17–20 base pair spacer located at the 5′-end of a single-guide RNA (sgRNA), a fusion of the crRNA and trRNA expressed as a single transcript [4,5]. To simultaneously induce breaks at several targets, multiple sgRNAs with different spacers, specific to each target, must be co-delivered. In plants, expression of Cas9 is typically driven by a strong constitutive promoter such as CaMV35S, while expression of the sgRNA is typically driven by a small nuclear RNA promoter utilising RNA polymerase III (Table 1).

**Table 1:** Regulatory and coding sequences used to express Cas9, Cpf1 and their corresponding gRNAs in different plant species The relative efficiencies obtained with different constructs cannot be readily compared due to the use of different genomic targets.

Species	Nuclease expression cassette			Promoter for guide RNA expression	Reference
	Promoter	Nuclease	Terminator		
*Arabidopsis thaliana* (Arabidopsis)	*Ubi*, *A. thaliana*	SpCas9, human CO	*Ubi*, *A. thaliana*	*U6-26*, *A. thaliana*; *U3b*, *A. thaliana*; *7SL-2*, *A. thaliana*	[64]
	35S, Cauliflower Mosaic Virus (CaMV)	SpCas9, human CO	*Nos*, *Agrobacterium tumefaciens*	*U6-26*, *A. thaliana*	[65]
	*35S*, *CaMV*;*WOX2*, *A. thaliana*; *RPS5A*, *A. thaliana*	SpCas9, human CO	*Hsp18.2*, *A. thaliana*	*U6-26*, *A. thaliana*	[66]
	*Ubi4-2*, *Petroselinum crispum*	SpCas9, *A. thaliana* CO	*rbcS3A*, *Pisum sativum*	*U6-26*, *A. thaliana*	[67]
	35S CaMV *enhancer fused to Z. mays C4PPDK basal promoter*	SpCas9, plant CO; SpCas9, human CO	*Nos*, *A. tumefaciens*	*U6-26*, *A. thaliana*	[45]
	*EC1.2*, *A. thaliana*	SpCas9, *Z. mays* CO	*rbcS-E9*, *P. sativum*	*U6-26*, *A. thaliana*	[68]
	*EC1.2*, *A. thaliana*	SpCas9, *Z. mays* CO	*rbcS-E9*, *P. sativum*; *Nos*, *A. tumefaciens*	*U6-26*, *A. thaliana*; *U6-29*, *A. thaliana*	[69]
	*Ubi10*, *A. thaliana*	SpCas9, *Z. mays CO*; SpCas9, *A. thaliana* CO	*Ocs*, *A. tumefaciens*	*U6-26*, *A. thaliana*	[70]
	2X35S, CaMV; *Ubi*, *P. crispum*; *DD45*, *A. thaliana*; *ICU2*, *A. thaliana*	SpCas9, human CO	*Nos*, *A. tumefaciens*; *Ocs*, *A. tumefaciens*	*U6-26*, *A. thaliana*	[71]
	*SPL*, *A. thaliana*; *Ubi*, *A. thaliana*; *DD45*, *A. thaliana*; *LAT52*, *Lyscopericum sculentum*	SpCas9, human CO	*SPL*, *A. thaliana*; *Ubi*, *A. thaliana*; *Nos*, *A. tumefaciens*	*U6-26*, *A. thaliana*	[72]
	*ICU2*, *A. thaliana*	SpCas9, human CO	*Nos*, *A. tumefaciens*	*U6-26*, *A. thaliana*	[73]
	*EC1.2*, *A. thaliana*; *MGE*, *A. thaliana*; *MGE2*, *A. thaliana*; *MGE3*, *A. thaliana*	SpCas9, *Z. mays* CO	*rbcS-E9*, *Pisum sativum*; *Nos*, *A. tumefaciens*	*U6-26*, *A. thaliana*	[74]
	2X35S, CaMV	SpCas9, human CO	*Nos*, *A. tumefaciens*	*U6-26*, *A. thaliana*; *U6-29*, *A. thaliana*; *U6-1*, *A. thaliana*	[21]
	2X35S, CaMV	SpCas9, plant CO	35S, CaMV	*U6-29*, *A. thaliana*; *U3*, *A. thaliana*	[22]
*Brassica oleracea*	Cassava Vein Mosaic Virus (CSVMV)	SpCas9, human CO	*Nos*, *A. tumefaciens*	*U6-26*, *A. thaliana*	[13]
*Citrullus lanatus* (Watermelon)	2x35S, CaMV	SpCas9, *Z. mays* CO	*Nos*, *A. tumefaciens*	*U6-26*, *A. thaliana*; *U6-29*, *A. thaliana*	[75]
*Citrus sinensis* (Sweet orange/Wanjincheng orange)	35S, CaMV	SpCas9, plant CO	*Nos*, *A. tumefaciens*	*U6-1*, *A. thaliana*	[76]
	35S, CaMV	SpCas9, human CO	*Nos*, *A. tumefaciens*	*35S*, *CaMV*	[77]
*Citrus paradisi* (Grapefruit)	35S, CaMV	SpCas9, human CO	*Nos*, *A. tumefaciens*	*35S*, *CaMV*	[78]
*Gossypium hirsutum* (Cotton)	2X35S, CaMV	SpCas9, *Z. mays* CO	*E9*	*U6-26*, *A. thaliana*; *U6-29*, *A. thaliana*	[79]
	2x35S, CaMV	SpCas9	*Nos*, *A. tumefaciens*	*U6-26*, *A. thaliana*	[80]
*Hordeum vulgare* (Barley)	*Ubi*, *Z. mays*	SpCas9	*Nos*, *A. tumefaciens*	*U6*, *Triticum aestivum*	[13]
	*Ubi*, *Z. mays*	SpCas9	*Nos*, *A. tumefaciens*	*U6*, *O. sativa*	[81]
*Marchantia polymorpha* L.	35S, CaMV; *EF1a*, *M. polymorpha*	SpCas9, human CO	*Not described*	*U6-1*, *M. polymorpha*	[82]
*Nicotiana tabacum*/*benthamiana*	35S CaMV *enhancer with the Z. mays C4PPDK basal promoter*	SpCas9, plant CO; SpCas9, human CO	*Nos*, *A. tumefaciens*	*U6-26*, *A. thaliana*	[45]
	2X35S, CaMV	SpCas9	*Nos*, *A. tumefaciens*	*U6-26*, *A. thaliana*	[83]
*Oryza sativa* (Rice)	*Ubi*, *Z. mays*	*AsCpf1*; *LbCpf1*	*Nos*, *A. tumefaciens*	*Ubi*, *Zea mays*	[39]
	*Ubi*, *Z. mays*; 2X35S, CaMV	SpCas9, plant CO	*Nos*, *A. tumefaciens*; *35S*, *CaMV*	*U3*, *O. sativa*; *U6b*, *O. sativa*; *U6c*, *O. sativa*	[22]
	2X35S, CaMV	SpCas9	35S, CaMV	*U3*	[84]
	2X35S, CaMV; *Ubi4-2*, *P. crispum*	SpCas9, human CO; SpCas9, *A. thaliana* CO; SpCas9, *O. sativa* CO	*rbcS3A*, *P. sativum*; *Nos*, *A. tumefaciens*; 35S, CaMV	*U6*, *O. sativa*; *U3*, *O. sativa*	[85]
	*Ubi*, *Z. mays*; 35S, CaMV	Nicking SpCas9 fused to cytosine deaminase; Nicking SpCas9, VQR mutation	*Nos*, *A. tumefaciens*	*U6*, *O. sativa*	[86]
	2X35S, CaMV	SpCas9 *O. sativa* CO; nuclease-deficient Cas9 D10A, H840A; nickase SpCas9 D10A; nickase SpCas9 H840A	*rbcS3A*, *P. sativum*	*U6*, *O. sativa*	[87]
	*Ubi*, *Z. mays*	LbCpf1, *O. sativa* CO	35S, CaMV	*U3*, *O. sativa*	[38]
	*Ubi*, *O. sativa*	SpCas9	*Nos*, *A. tumefaciens*	*U3*, *O. sativa*	[88]
*Petunia hybrid* (Petunia)	35S, CaMV	SpCas9, plant CO; SpCas9, human CO	*Nos*, *A. tumefaciens*; 35S, CaMV	*U6-26*, *A. thaliana*	[89]
*Physcomitrella patens*	*EF1a*, *Scopelophila cataractae*	SpCas9, Fungus CO; SpCas9, plant CO	*TrbcS*, *S. cataractae*	*U6*, *S. cataractae*	[90]
	*Act1*, *O. sativa*	SpCas9, human CO	*Nos*, *A. tumefaciens*	*U6*, *P. patens*; *U3*, *P. patens*	[91]
*Populus* (Poplar)	35S, CaMV	SpCas9, human CO	*Not described*	*U6*, *Medicago truncatula*	[92]
	2X35S, CaMV	SpCas9, plant CO	35S, CaMV	*U6-1*, *A. thaliana*; *U6-29*, *A. thaliana*; *U3b*, *A. thaliana*; *U3d*, *A. thaliana*	[93]
*Salvia miltiorrhiza* (red sage)	2X35S, CaMV	SpCas9	*Not described*	*U6-26*, *A. thaliana*	[94]
*Scopelophila cataractae* (tongue-leaf copper moss)	*EF1a*, *S. cataractae*	SpCas9, plant CO	*TrbcS*, *S. cataractae*	*U6*, *S. cataractae*	[90]
*Solanum lycopersicum* (Tomato)	35S, CaMV	SpCas9, human CO	*Nos*, *A. tumefaciens*	*U6-26*, *A. thaliana*	[26]
	*Ubi*, *P. crispum*	SpCas9, *A. thaliana* CO; nickase SpCas9 D10A	*pea rbcS3A*, *P. sativum*	*U6-26*, *A. thaliana*	[87]
*Solanum tuberosum* (Potato)	2x35S, CaMV	SpCas9, *O. sativa* CO	*Nos*, *A. tumefaciens*	*U6*, *S. tuberosum*	[95]
	2x35S, CaMV	SpCas9, *A. thaliana* CO	*Nos*, *A. tumefaciens*	*U6-26*, *A. thaliana*	[46]
	2x35S, CaMV; *Ubi*, *O. sativa*	SpCas9, *Z. mays* CO	*Nos*, *A. tumefaciens*	*U3*, *O. sativa*; *U6*, *O. sativa*; *U3*, *T. aestivum*	[96]
	35S, CaMV	SpCas9, plant CO	*Nos*, *A. tumefaciens*	*U6-26*, *A. thaliana*; *U6*, *S. tuberosum*	[97]
	*Ubi*, *Zea mays*	SpCas9, *O. sativa* CO	*Nos*, *A. tumefaciens*	*U6-2*, *O. sativa*	[98]
*Triticum aestivum* (Wheat)	*Ubi1*, *Zea mays*	SpCas9, *T. aestivum* CO	*Not described*	*U6*, *T. aestivum*	[42]
	*Ubi-1*, *Zea mays*	SpCas9	35S, CaMV	*U6*, *T. aestivum*	[99]
*Zea mays* (maze)	*Ubi*, *Zea mays*	SpCas9, *Z. mays* CO	*Nos*, *A. tumefaciens*	* U6*, *Z. mays*	[100]
	2x35S, *CaMV*; *Ubi*, *O. sativa*	SpCas9, *Z. mays* CO	*Nos*, *A. tumefaciens*	*U3*, *O. sativa*; *U6*, *O. sativa*; *U3*, *T. aestivum*	[21]
	*Ubi*, *Z. mays*	SpCas9, *Z. mays* CO	*Nos*, *A. tumefaciens*	*U6*, *Z. mays*	[101]
	2x35S, CaMV	SpCas9	35S, CaMV	*U3*, *Z. mays*	[102]
	2x35S, CaMV	SpCas9, *Z. mays* CO	*Nos*, *A. tumefaciens*	*U3*, *Z. mays*	[103]
	*Ubi*, *Z. mays*	SpCas9, *O. sativa* CO	*Ocs*, *A. tumefaciens*	*U6-1*, *Z. mays*; *U6-2*, *Z. mays*	[104]

Abbreviation: CO, codon optimised.

The typical workflow for obtaining plants with targeted mutations at one or more loci is to deliver a construct comprising a selectable marker gene; a Cas9 expression cassette and one or more sgRNA cassettes to plant tissues by an established DNA-delivery method. In many species, this is mediated by *Agrobacterium tumefaciens*, aiming for stable integration of transgenes into the plant genome [6]. The transgenic plants are recovered on selection and assayed for mutations at the target locus (or loci), generally small insertion or deletion events. Most studies report the percentage of transgenic plants in which mutations are found as the ‘efficiency’ of targeted mutagenesis. This varies both between genomic targets and between species. If mutations are not induced in the cells from which a transgenic plant regenerates (often the cells of callus tissue), but instead occur during the growth and development of the regenerating plant, different repair outcomes of multiple double-strand breaks (DSBs) in each cell or cell line will lead to a genetically chimeric plant [7–10]. However, many studies have reported the recovery of plants with homozygous or biallelic mutations (a different mutation in each homologous chromosome) in the first generation [9,10]. Transgenes delivered by *A. tumefaciens* are generally randomly inserted into the genome, are hemizygous in the regenerated generation (known as ‘T0’) and are often at low copy number. In many species, it has therefore been possible to segregate the transgene locus and the target locus in the progeny (or T1) generation of at least some transgenic events, resulting in transgene-free plants with mutations at the desired target. This has been demonstrated in many species, for example *Arabidopsis thaliana* (Arabidopsis) [11], *Oryza sativa* (rice) [12], *Hordeum vulgare* (barley) [13] and *Brassica oleracea* [13].

## Toolkits for targeted mutagenesis

**Figure 1.**
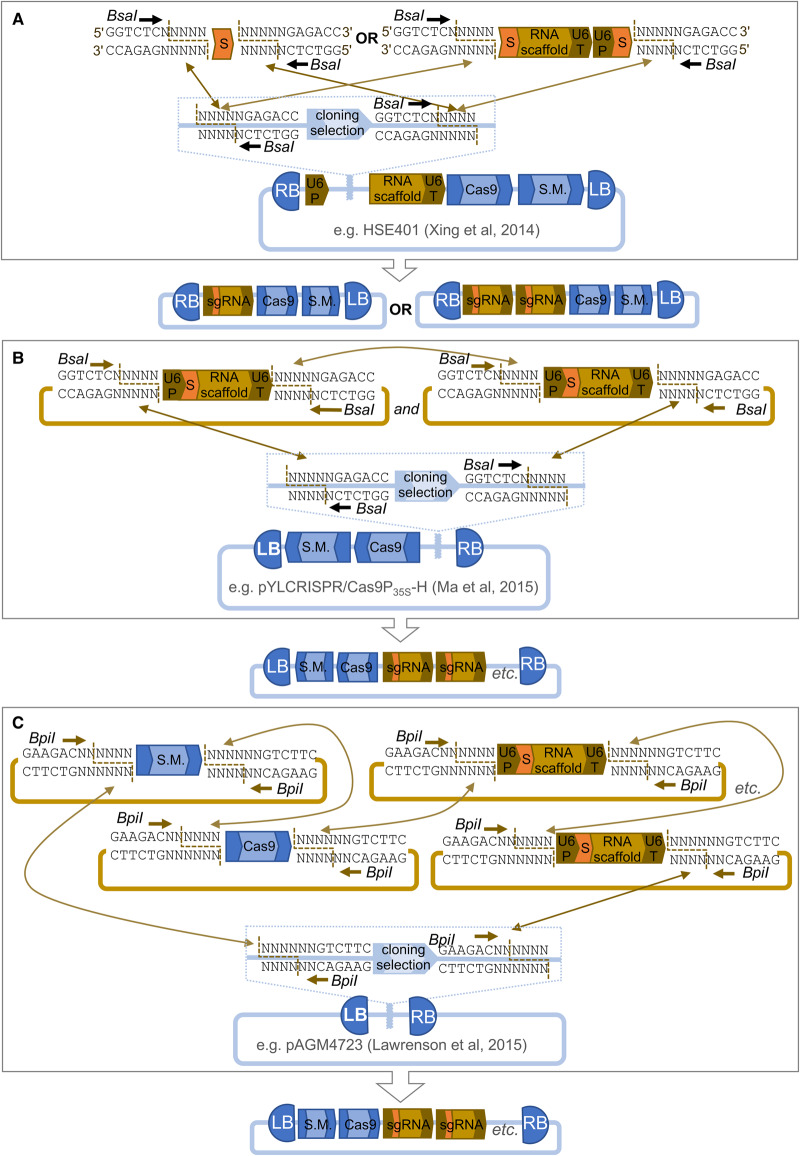
Many toolkits utilise Type IIS restriction enzymes to assemble constructs with multiple sgRNAs for multiplexed Cas9-mediated targeted mutagenesis. Type IIS enzymes such as *BsaI* cut outside of their recognition sequences in regions with no sequence requirements, shown as ‘NNNN’. This allows multiple fragments to be assembled simultaneously in a selected order by choosing unique sequences for each fusion site. (**A**) Xing et al. [21] constructed a series of binary backbones with different Cas9 and plant selectable marker cassettes (S.M.). The spacer sequence (S) for a gene-specific target can be added to a pair of convergent *BsaI* sites between a U6 promoter (U6P) and the RNA scaffold/U6 terminator (U6T), completing the sgRNA cassette. Alternatively, two or more sgRNA cassettes are assembled by PCR and the amplicon inserted at the same site. (**B**) Ma et al. [22] constructed a series of backbones with pairs of convergent *BsaI* sites into which multiple sgRNA cassettes can be assembled. Gene-specific spacers are first added to the sgRNA cassettes by overlap PCR. (**C**) Nekrasov et al. [25] and Lawrenson et al. [13] utilised the MoClo plasmid toolkits adding Cas9 and sgRNA standard parts to the existing part sets. Gene-specific spacers are first assembled into sgRNA cassettes by PCR and then multiple sgRNA cassettes are assembled into a binary backbone together with Cas9 and S.M. cassettes appropriate for the species of interest in a single cloning reaction.

To allow both the simultaneous targeting of multiple genes and the segregation of transgenes from the target locus (or loci), it is highly desirable for all gene cassettes to be delivered together in a single multigene vector so that all elements integrate at a single genetic locus. Previously, the assembly of complex multigene constructs was considered a bottleneck in biotechnology. However, concurrent with the emergence of molecular tools for genome-editing, several new methods that enable the facile parallel (simultaneous) assembly of multiple DNA parts with minimal scars have emerged from the nascent field of synthetic biology [14,15]. The most widely adopted of these are Type IIS restriction endonuclease-mediated assembly, widely known as Golden Gate Cloning [16–18], and a ligation-independent method that requires the production of linear overlapping fragments known as Gibson Assembly [19,20]. In particular, many plasmid toolkits utilising a Type IIS restriction enzyme, frequently *BsaI*, have been created to facilitate the simultaneous assembly of multiple sgRNAs to allow simultaneous induction of mutations at multiple genomic targets, sometimes referred to as multiplexed mutagenesis (Figure 1). A sgRNA may also be designed to recognise more than one target locus, for example in closely related gene families.

Broadly, three approaches have been used to simplify the construction of multigene constructs for Cas9-mediated targeted mutagenesis in plants. The first, employed by Xing et al. [21] for targeted mutagenesis in *Zea mays* (maize) and Arabidopsis, is to create plasmid backbones containing a selectable marker, Cas9 expression and sgRNA expression cassettes, with regulatory sequences, e.g. promoters and terminators, appropriate for the plant species of interest. The spacer is then inserted into the sgRNA cassette using a Type IIS enzyme to enable scarless cloning (no additional nucleotides introduced between the assembled fragments; Figure 1A). To introduce additional sgRNA cassettes for multiplexed mutagenesis, a PCR amplicon comprising one or more additional cassettes is inserted into the same cloning site. The advantage of this system is that, once the initial plasmid is constructed for the species of interest, only a single cloning reaction is required. The disadvantage is that a new, bespoke plasmid construct must be engineered for each species.

A second approach, exemplified by the toolkits created by Ma et al. [22] and Lowder et al. [23], is to create plasmid backbones containing a selectable marker and Cas9 expression cassettes suitable for the species of interest, as well as a second set of plasmids containing individual sgRNA cassettes (Figure 1B). In the first cloning step, spacers are inserted either by PCR [22] or by using a Type IIS enzyme [23]. In the second step, one or more sgRNA cassettes are simultaneously assembled into the final delivery backbone using either multisite Gateway® Cloning [23] or Type IIS-mediated assembly [22]. Ma et al. demonstrated the assembly of constructs with up to eight sgRNA expression cassettes collectively recognising a total of 46 target loci in rice. These systems are shown to be easily applicable for multiplexed targeted mutagenesis, but also require the engineering of a bespoke backbone for each species.

The final approach uses existing Type IIS assembly plasmid toolkits such as the Golden Gate Modular Cloning (MoClo) toolkit [17] and GoldenBraid (GB) [24]. The use of these flexible toolkits for Cas9-mediated targeted mutagenesis has been demonstrated in several species: the MoClo toolkit has been utilised in *Nicotiana benthamiana* [25], *Solanum lycopersicum* (tomato) [26] and barley (Figure 1C) [13], and the GB toolkit has been demonstrated *in N. benthamiana* [27]. The flexibility of these toolkits allows for any type and number of cassettes to be assembled making it easy to include any number of sgRNA cassettes. Both the MoClo and GB plasmid toolkits are modular and hierarchical. In the first step, standard DNA parts (e.g. promoters, coding sequences or sgRNA scaffolds and terminators) are assembled into gene expression cassettes in a single step using the Type IIS enzyme *BsaI*. These gene expression cassettes can then be assembled into multigene constructs using a second Type IIS enzyme (Figure 1C). Čermák et al. [28] also created a binary plasmid backbone into which gene expression cassettes can be simultaneously assembled using the Type IIS enzyme *AarI*. The advantage of these toolkits is that the interoperable, modular parts can be reused in new assemblies with equal simplicity making application to new species, as well the inclusion of variant parts easy to implement.

To avoid the necessity for multigene constructs and to increase the expression level of the sgRNA, viral delivery vectors have been used to transiently express sgRNAs in plants overexpressing Cas9 from a stably integrated transgene. Both Tobacco Rattle Virus (TRV) [29] and Cabbage Leaf Curl Virus (CaLCuV) [30] have been adapted for this purpose. More recently, Cody et al. [31] demonstrated targeted mutagenesis induced by transient expression of Cas9 simultaneous with delivery of sgRNAs from a tobacco mosaic virus-derived vector.

As described above, most efforts at multiplexed targeted mutagenesis have focused on tools for facile assembly of multiple sgRNA cassettes. However, polycistronic mRNAs with multiple sgRNAs have also been used to avoid the necessity for an individual promoter for each sgRNA. Xie and Yang [32] expressed a polycistronic transcript of two sgRNAs separated by transfer RNA (tRNA) sequences. The tRNA sequences are cleaved by endogenous tRNA-processing RNases to release the individual sgRNAs. Čermák et al. [28] compared polycistronic transcripts of multiple sgRNAs separated by either tRNAs, self-cleaving ribozymes or recognition sequences for Csy4, a ribonuclease expressed in translational fusion with Cas9 separated by the self-cleaving 2A peptide from porcine teschovirus 1. Csy4 was found to be the most efficient and was used to express a polycistronic transcript comprising six sgRNAs in *Medicago trunculata*, successfully recovering plants in which a 58 kb genomic fragment had been deleted [28].

**Figure 2.**
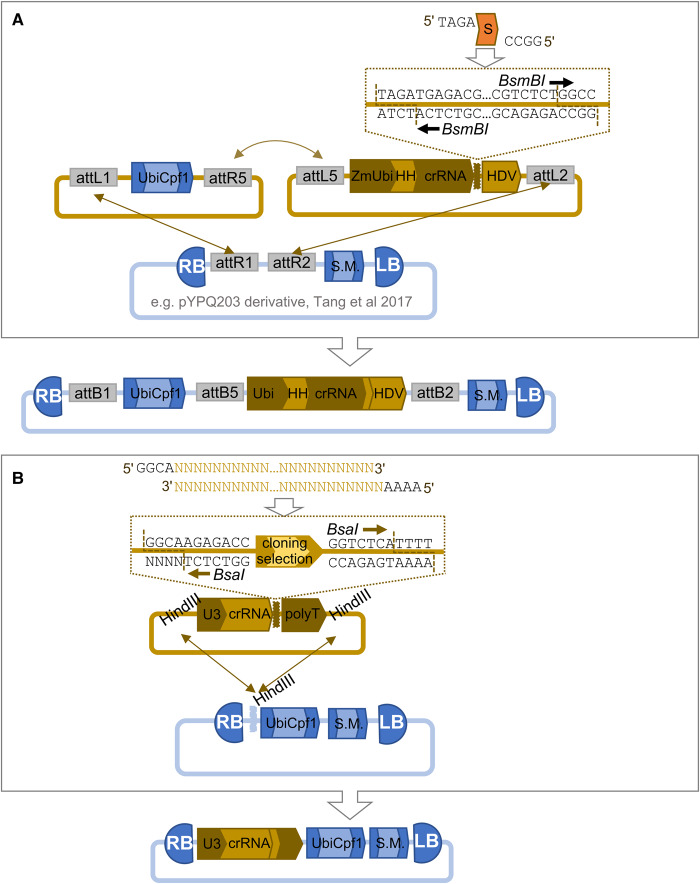
Plasmids for Cpf1-mediated targeted mutagenesis in plants. (**A**) Tang et al. [39] used Multisite Gateway cloning (recombination sites are annotated as attL and attR) to assemble a Cpf1 expression cassette (UbiCpf1) and a CRISPR RNA (crRNA) cassette into a binary backbone containing a plant selectable marker cassette (S.M.). The crRNA was flanked by the Hammerhead (HH) and Hepatitis Delta Virus (HDV) ribozyme sequences to self-process after transcription from a constitutive RNA polymerase II promoter (Ubi). Gene-dependent spacer sequences (S) were made by cloning annealed, phosphorylated oligonucleotides into a pair of divergent *BsmBI* sites. (**B**) Xu et al. [38] constructed backbones containing Cpf1 and S.M. cassettes and added crRNA cassettes to an *HindIII* site. Expression of the crRNA was driven by an RNA polymerase III-dependent U3 promoter (U3) and a gene-specific spacer was added by cloning annealed, phosphorylated oligonucleotides into a pair of divergent *BsaI* sites.

The majority of studies have utilised the wild-type Cas9 from *Streptococcus pyogenies* (SpCas9), which recognises the canonical NGG PAM. However, variants of SpCas9 with mutations in the PAM-recognition domain that enable recognition of NGA PAMs [33] have been used to induce mutagenesis in the rice genome [34], and Cas9 proteins from other species have also been demonstrated to function in plants, for example Cas9 from *Staphylococcus aureus* [35,36]. Recently, Cpf1 nucleases found in the CRISPR systems of *Francisella novicida* (FnCpf1), *Lachnospiraceae bacterium* ND2006 (LbCpf1) and *Acidaminococcus* sp. BV3L6 (AsCpf1) were adopted for genome engineering in eukaryotes [37]. FnCpf1 recognises a TTN PAM, while LbCPf1 and AsCpf1 prefer a TTTV PAM [37]. Xu et al. [38] constructed tools for Cpf1-mediated mutagenesis in rice by constructing a plasmid backbone containing a selectable maker and Cpf1 expression cassettes. Spacer sequences were inserted into the crRNA that guides Cpf1 to the target by annealing a phosphorylated oligonucleotide dimer into a Type IIS cloning site before assembly into the final plasmid (Figure 2A). In contrast, Tang et al. [39] flanked the crRNA with self-cleaving ribozymes enabling them to drive expression in rice from a strong constitutive promoter (Figure 2B). Notably, Tang et al. [39] reported 100% mutation efficiency, with very few plants being genetic chimeras.

## Tools for targeted insertion

**Figure 3.**
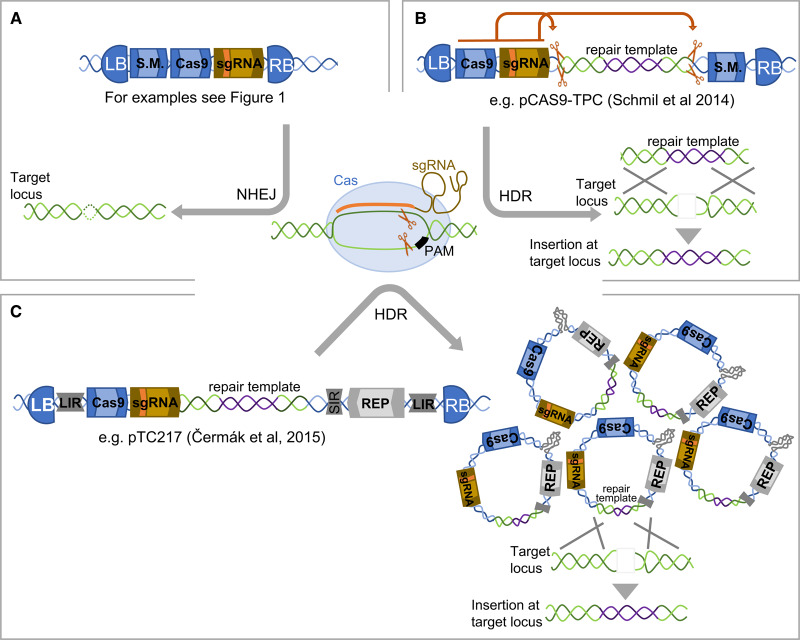
Comparison of plasmid features used for Cas9-mediated targeted mutagenesis and site-specific insertion in plants. In all cases, the sgRNA and Cas protein typically induce a double-stranded break three bases pairs upstream of the PAM. The frequently used wild-type Cas9 from *S. pyogenes* recognises an NGG PAM. (**A**) In the absence of any repair template other than the sister chromatid, repair of the break occurs through the, sometimes imperfect, NHEJ repair pathway and can result in a small insertion, deletion or rearrangement. Additional plasmid features can be added to influence the outcome of Cas9-induced DNA-break repair: (**B**) The inclusion of repair template with homology to the regions flanking the cut-site is included as a template for HDR. Schiml et al. [43] used this strategy for targeted gene insertion releasing a linear repair template by the inclusion of sgRNA recognition sites flanking the homologous regions. (**C**) To increase the amount of repair template available for HDR, Baltes et al. [41] utilised a geminiviral replicon. They flanked the Cas9, sgRNA cassettes and repair template with large and small intergenic regions (LIR and SIR) and replicase (REP) to enable the production of large quantities of circular double-stranded DNA in the nucleus.

The induced mutations mediated by CRISPR-associated proteins are the result of imperfect repair of DSBs, typically by the endogenous mechanism of non-homologous end-joining (NHEJ; Figure 3A). Most reports of targeted insertion of DNA at induced DSBs have sought to use homology-directed repair (HDR) — for a recent review of plant DNA repair mechanisms see [40]. HDR uses a template with homology to the sequence in which the DSB has been induced and is therefore less likely to induce errors. HDR can be used either for targeted insertion of new DNA or to recode, edit or replace an endogenous sequence. To achieve this, repair templates containing the sequence for insertion or editing are flanked by regions of homology to the regions adjacent to the targeted DSB. This repair template is co-delivered with the nuclease expression cassette(s) and guide or CRISPR RNA (Figure 3B) [28,41–45].

One of the challenges of HDR in plants is the delivery of sufficient quantities of repair template concurrent with the creation of the DSB. Viral replicons based on Bean Yellow Dwarf Virus (BeYDV), Tomato Leaf Curl Virus (ToLCV) and Wheat Dwarf Virus (WDV) have been used to increase the number of copies of the repair template in many plant species, successfully increasing the frequency of targeted DNA insertion up to 10-fold [28,41,42,46,47]. The key elements of the geminiviral replicon are a large intergenic region (LIR), a small intergenic region (SIR) and overlapping coding sequences for the Rep and RepA proteins required for replication [41,47]. All sequences cloned between the LIR and SIR will be amplified on a circular, double-stranded DNA replicon, which accumulates at high copy in the nucleus (Figure 3C).

## Tools for modulation of gene expression

**Figure 4.**
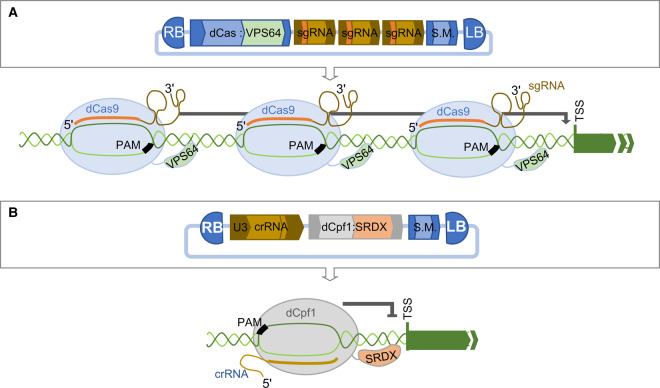
dCas9 and Cpf1 proteins are used to locate effector domains including transcriptional activators and repressors to the regulatory regions of endogenous genes. (**A**) Lowder et al. [23] assembled a multigene construct comprising a cassette expressing Cas9 mutated to removed nuclease activity (dCas9) fused to a transcriptional activator domain (VPS64) and three sgRNA cassettes. Each sgRNA contained a spacer to direct the Cas9 : VPS64 fusion protein to sequences immediately upstream of the transcriptional start site (TSS) of an endogenous gene to up-regulate gene expression. (**B**) Xu et al. [38] fused the coding sequence of Cpf1 mutated to remove nuclease activity (dCpf1) to a transcriptional repressor domain (SRDX). A crRNA directed the dCpf1 : SDRX fusion protein to a sequence proximal to the TSS of an endogenous gene to demonstrate a reduction in gene expression.

Cas9 has two nuclease domains, HNH and RuvC. Disruption of both domains results in deactivated Cas9 (dCas9), which has no nuclease activity but retains the ability to form a duplex with a sgRNA and to scan genomic DNA for PAM motifs allowing the sgRNA to pair with its cognate sequence [48]. dCas9 can be fused to effector domains such as transcriptional activators and repressors to modulate expression of target genes [48]. Lowder et al. [23] demonstrated tuneable activation of transcription in plants by fusing dCas9 with the well-known transcriptional activator domain, VPS64. Three sgRNAs were designed to recognise the promoter of the target gene and assembled with dCas9 : VPS64 and selectable marker cassettes (Figure 4A). Tang et al. [39] demonstrated the use of Cpf1 for transcriptional control in Arabidopsis by fusing an SRDX transcriptional repressor domain to LbCpf1 and AsCpf1 with disrupted nuclease domains (Figure 4B). They successfully reduced expression of the target gene to 10% of wild type.

Several additional methods have been demonstrated in non-plant systems to co-recruit multiple activator domains to the same locus, thus increasing the level of transcriptional activation. A repeated peptide array, known as Suntag, was fused to dCas9 to recruit multiple VPS64 activators bound to single-chain variable fragments to the same locus [49,50]. In another study, a so-called Synergistic Activation Mediator system co-expressed dCas9 : VPS64, an sgRNA modified to contain two MS2 aptamers, and the RNA-binding MS2 coat protein fused to the transcriptional activator p65 and the activating domain of heat shock protein factor 1 [50,51]. In other experiments performed in mammalian and human cell lines, dCas9 has been fused to catalytic domains for epigenetic engineering. In one study, dCas9 was fused to the catalytic core of the acetyltransferase, p300, to acetylate histone H3 in promoter sequences leading to transcriptional activation [52]. To repress transcription, dCas9 was fused to the Krüpel-associated box involved in recruiting a heterochromatin-forming complex and also to the lysine-specific demethylase 1 histone demethylase, which catalyses the removal of methyl marks on histone H3K4 and H3K9 [53]. Targeting of dCas9 fused to ten-eleven translocation methylcytosine dioxygenase 1 (TET1) or the catalytic domain of the DNA methyltransferase, DNMT3A, to methylated or unmethylated promoter sequences caused activation or silencing, respectively [54,55]. DNA methylation induced by targeted DNMT3A activity was observed to be specific for the targeted region and heritable across mitotic divisions [54].

## Tools for DNA-free engineering

**Figure 5.**
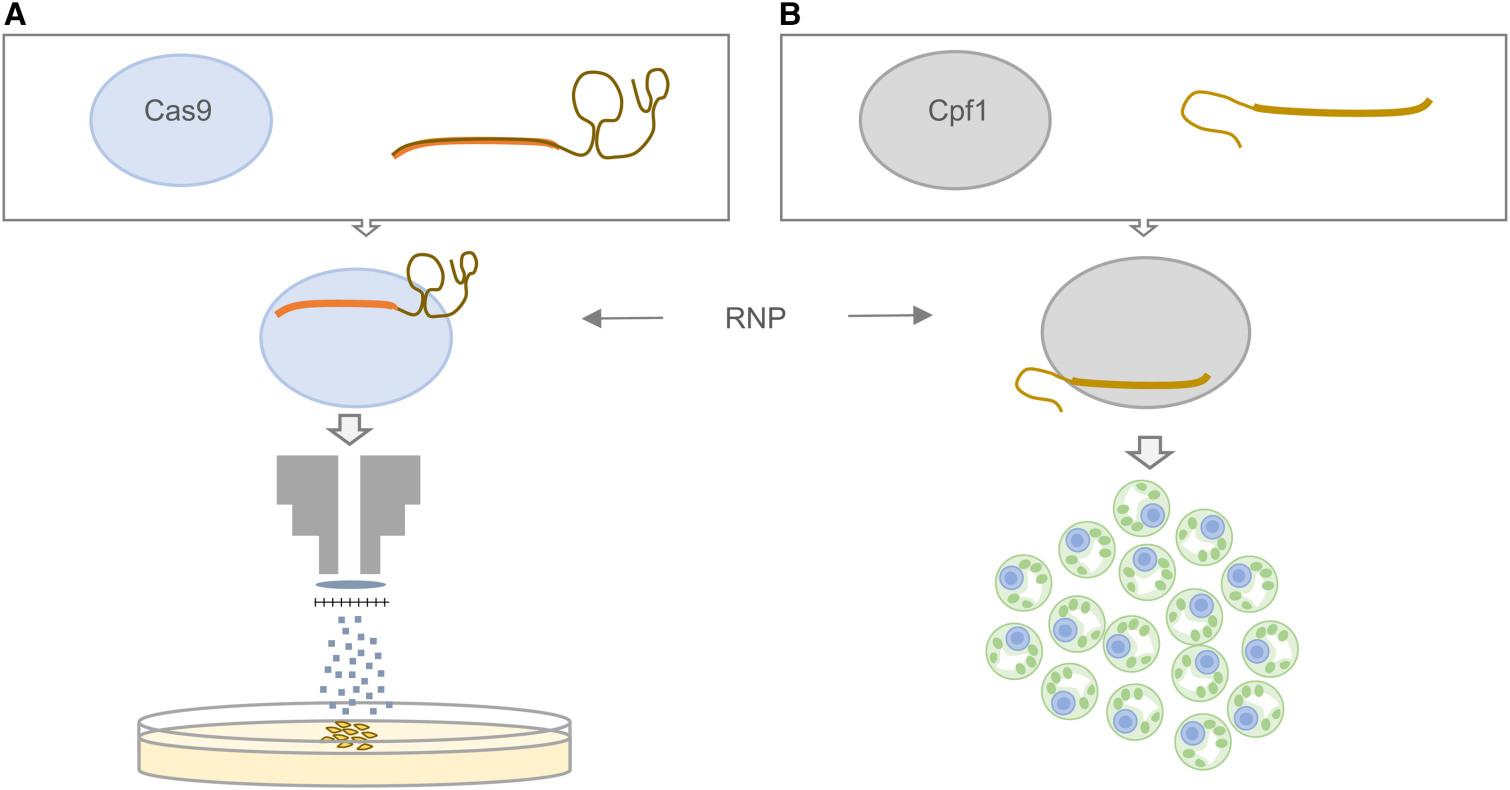
Production of Cas9 and Cpf1 RNPs for DNA-free targeted mutagenesis. Both Cas9 and CPf1 nucleases can be purchased commercially or produced by heterologous expression in *E. coli.* Bespoke single sgRNAs and crRNAs can either be purchased or produced by *in vitro* transcription from a PCR amplicon into which a T7 promoter is introduced in the forward primer. RNP complexes have been used to induce targeted mutagenesis in plant species including (**A**) wheat by biolistic delivery of Cas9 RNPs to immature embryos [60] and (**B**) soybean and wild tobacco by delivery of Cpf1 RNPs to protoplasts [61].

Direct delivery of the Cas9 or Cpf1 protein in complex with the guide RNA, known as the ribonuclease (RNP) complex, avoids the introduction of DNA into the cell. RNP-mediated genome engineering was first shown in mammalian cells [56,57] but has since been demonstrated in many plant species [58–61]. Purified Cas9 protein is commercially available or can be overexpressed in *Escherichia coli* (Figure 5). Similarly, RNA moieties can be purchased or produced by *in vitro* transcription. RNP complexes have been delivered to plant tissues using particle bombardment (Figure 5A) and also by direct delivery to protoplasts (Figure 5B). Woo et al. [58] transfected Cas9 RNPs into protoplasts of Arabidopsis, *Nicotiana tabacum* (tobacco), *Lactuca sativa* (lettuce) and rice. Mutations at the target were found in 46% of callus tissues regenerated from lettuce protoplasts. Kim et al. [61] used *LbCpf1* and *AsCpf1* RNPs to induce targeted mutagenesis in protoplasts of soybean and tobacco (Figure 5B). Svitashev et al. [59] and Liang et al. [60] delivered RNPs into embryo cells of maize and wheat, respectively, using particle bombardment. Although reported to be less efficient, the use of RNPs has many advantages. Firstly, RNP-mutated plants are reported to have less mutations at off target-sites, presumably because the protein complex does not persist throughout plant development and therefore has fewer opportunities to induce DSBs [60]. Secondly, there is no requirement for segregation of a transgene. Finally, as the plants have never had foreign DNA introduced into the genome, it is reasonable to speculate that they may not be subject to same extensive regulatory processes as plants made using a transgenic approach. In some administrations, where the process used to produce a plant with a desired genotype triggers the regulatory process, this may be relatively more advantageous than in nations where only the end product is evaluated [58–62].

## Conclusions

The CRISPR system has provided several reliable, flexible and robust methods for engineering plant genomes. The construction of molecular tools to enable their use in eukaryotes, including plans, has been facilitated by parallel assembly methods that allow multiple fragments of DNA to be assembled together in a single cloning reaction. Mutagenesis of the Cas9 nuclease and the adoption of new tools such as Cpf1 have removed the limitation of wild-type SpCas9 to targets associated with NGG PAMs, providing researchers with the opportunity to mutate or deliver protein cargos such as transcriptional activators to a much wider number of sites across plant genomes. Targeted insertion and the editing of endogenous gene to a desired sequence remains challenging, however, targeted mutagenesis has been shown to be efficient in many plant species (Table 1), enabling experimental strategies previously limited to the few model species for which knockout libraries exist. Studies are rapidly progressing from proof of concept to applications in research and crop improvement providing a wealth of new opportunities. The inheritance of induced mutations in the absence of a transgene poses new questions for the regulation of engineered plants, especially food crops, for which criteria have previously focused on the sequence and genetic location of integrated DNA [62,63].

## Summary

- Molecular tools adapted from bacterial CRISPR systems have been applied for the induction of targeted mutations in many plant species.
- The construction of molecular tools for genome engineering has been facilitated by techniques for parallel DNA assembly.
- Modifications of Cas9 and the exploitation of new CRISPR systems allow targeted engineering of an increasing number of genomic targets.
- The direct delivery of protein–RNA complexes to plant cells avoids the introduction of DNA into the genome.

## Abbreviations

Cas: CRISPR-associated
CRISPR: clustered regulatory interspaced short palindromic repeats
crRNA: CRISPR RNA
dCas9: deactivated Cas9
DSBs: double-strand breaks
GB: GoldenBraid
gRNAs: guide RNAs
HDR: homology-directed repair
LIR: large intergenic region
MoClo: Modular Cloning
NHEJ: non-homologous end-joining
PAMs: protospacer adjacent motif
RNP: ribonuclease
sgRNA: single-guide RNA
SIR: small intergenic region
tRNA: transfer RNA
trRNA: *trans*-activating RNA
